# Selection for high levels of resistance to double-stranded RNA (dsRNA) in Colorado potato beetle (*Leptinotarsa decemlineata* Say) using non-transgenic foliar delivery

**DOI:** 10.1038/s41598-021-85876-1

**Published:** 2021-03-22

**Authors:** Swati Mishra, James Dee, William Moar, Jodi Dufner-Beattie, James Baum, Naymã Pinto Dias, Andrei Alyokhin, Aaron Buzza, Silvia I. Rondon, Mark Clough, Sandy Menasha, Russell Groves, Justin Clements, Ken Ostlie, Gary Felton, Tim Waters, William E. Snyder, Juan Luis Jurat-Fuentes

**Affiliations:** 1 Department of Entomology and Plant Pathology, University of Tennessee, Knoxville, TN 37996 USA; 2 Bayer Crop Science, 700 Chesterfield Pkwy West, FF4336B, Chesterfield, MO 63017 USA; 3 School of Biology and Ecology, University of Maine, Orono, ME 04469 USA; 4 Department of Botany and Plant Pathology, Hermiston Agricultural Research and Extension Center, Oregon State University, Hermiston, OR 97838 USA; 5 Vernon G. James Research and Extension Center, North Carolina State University, 207 Research Station Rd., Plymouth, NC 27962 USA; 6 Cornell Cooperative Extension, Cornell University, Suffolk County, Riverhead, NY 11901 USA; 7 Department of Entomology, University of Wisconsin-Madison, Madison, WI 53706 USA; 8 Department of Entomology, University of Minnesota, St. Paul, MN 55108 USA; 9 Department of Entomology, The Pennsylvania State University, University Park, PA 16802 USA; 10 Washington State University, 404 W. Clark Avenue, Pasco, WA 99301 USA; 11 Department of Entomology, University of Georgia, Athens, GA 30602 USA

**Keywords:** Entomology, RNAi

## Abstract

Insecticidal double-stranded RNAs (dsRNAs) silence expression of vital genes by activating the RNA interference (RNAi) mechanism in insect cells. Despite high commercial interest in insecticidal dsRNA, information on resistance to dsRNA is scarce, particularly for dsRNA products with non-transgenic delivery (ex. foliar/topical application) nearing regulatory review. We report the development of the CEAS 300 population of Colorado potato beetle (*Leptinotarsa decemlineata* Say) (Coleoptera: Chrysomelidae) with > 11,100-fold resistance to a dsRNA targeting the V-ATPase subunit A gene after nine episodes of selection using non-transgenic delivery by foliar coating. Resistance was associated with lack of target gene down-regulation in CEAS 300 larvae and cross-resistance to another dsRNA target (COPI β; Coatomer subunit beta). In contrast, CEAS 300 larvae showed very low (~ 4-fold) reduced susceptibility to the Cry3Aa insecticidal protein from *Bacillus thuringiensis*. Resistance to dsRNA in CEAS 300 is transmitted as an autosomal recessive trait and is polygenic. These data represent the first documented case of resistance in an insect pest with high pesticide resistance potential using dsRNA delivered through non-transgenic techniques. Information on the genetics of resistance and availability of dsRNA-resistant *L. decemlineata* guide the design of resistance management tools and allow research to identify resistance alleles and estimate resistance risks.

## Introduction

Publication of the first study providing experimental evidence for the use of insecticidal double-stranded RNA (dsRNA) as a plant-incorporated protectant^1^ led to increasing interest in the commercial development of this technology for pest control^2^. Uptake of the ingested dsRNA into the gut cells of targeted insects triggers the RNA interference (RNAi) response, initiated by cleavage of the dsRNA into 21–25 bp small interfering RNAs (siRNAs). These siRNAs are then loaded into the RNA-induced silencing complex (RISC) and unwound to yield a guide strand complementary to the target mRNA^3^. The guide strand recognizes the target transcript, triggering degradation to effectively reduce target gene expression. Targeting transcripts of essential genes via RNAi can result in inhibition of growth^4^, developmental abnormalities^5^, and/or mortality^1^. The high specificity of RNAi, driven by sequence identity between the guide strand and the mRNA, makes it a preferable tool over broad-spectrum insecticides, protecting beneficial and non-target organisms^6^ and fits well with integrated pest management (IPM) efforts. In addition, dsRNA represents a unique mechanism of action compared to bacterial insecticidal proteins present in sprayable formulations and expressed in transgenic crops, suggesting their combined use to delay evolution of resistance^7,8^.

Interest in commercialization of this technology has fueled progress towards evaluating the insecticidal activity of dsRNA against several insect pests, and studying the underlying mechanisms that determine RNAi efficiency, including different methods of dsRNA delivery^9^. Currently, transformative (i.e., transgenic plants) and non-transgenic (foliar sprays, root drenches and trunk injections) dsRNA delivery approaches are being considered for insecticidal dsRNA products. The first transgenic event including dsRNA as a plant incorporated protectant in addition to Cry3Bb1 and Cry34Ab1/35Ab1 (reassigned as Gpp34Ab1/Tpp35Ab1 by the Bacterial Pesticidal Protein Resource Center, BPPRC)^10^ insecticidal proteins from *Bacillus thuringiensis* (SmartStax Pro maize), was approved by the US Environmental Protection Agency in 2017 for control of rootworms (*Diabrotica* sp)^11^. Non-transgenic foliar application of dsRNA directed against the Colorado potato beetle (*Leptinotarsa decemlineata* Say)^12^ represents a second delivery method expected to undergo regulatory registration in 2020 (https://www.greenlightbiosciences.com/application/stopping-the-colorado-potato-beetle/).

To ensure the durability of insecticidal dsRNA technology, it is essential to develop effective insect resistance management (IRM) strategies. Critical in guiding development of these IRM plans is knowledge of the mechanisms and genetics of resistance to dsRNA. Information on resistance mechanisms can also aid in further optimization of the technology. The only currently available evidence for a mechanism of resistance to dsRNA in an insect relates to a field strain of *Diabrotica virgifera virgifera* selected with transgenic maize producing a dsRNA targeting the *D. v. virgifera* Snf7 (*DvSnf7*) gene^8^. Resistance (> 130-fold) in this case was recessive, localized to a single locus and associated with impaired dsRNA uptake into midgut cells. Importantly, dsRNA-resistant rootworms were not cross-resistant to Cry3Bb1, confirming a previous report of lack of cross-resistance to dsRNA in Cry3Bb1-resistant *D. v. virgifera*^7^, supporting the pyramiding of dsRNA with Cry toxins to combine different modes of action and delay evolution of resistance.

In contrast to transgenic delivery of dsRNA, there is currently no available information for any insect on mechanisms of resistance to insecticidal dsRNA delivered using non-transgenic methods. The main differences between the transgenic versus non-transgenic delivery methods could be the ability to control dosage and dsRNA wash-off in foliar dsRNA sprays resulting in a less than optimal dose. On the other hand, exposure to relatively high concentrations of dsRNA in spray applications could result in selection of major resistance alleles that may not be selected as efficiently by lower RNA concentrations as observed in *D. v. virgifera* with DvSnf7^8^. In addition, it is plausible that different mechanisms of resistance to dsRNA may be selected in different target species, as suggested by the differences in susceptibility to dsRNA observed among insects^13,14^. These observations highlight the importance of considering different delivery methods and target pests in dsRNA resistance studies.

To resolve this knowledge gap, we selected and characterized resistance in *L. decemlineata* to insecticidal dsRNA delivered using a non-transgenic approach akin to foliar spraying. This chrysomelid is an economically-relevant pest of solanaceous crops with a notorious ability for evolving resistance to pesticides^15^ and is a major target of dsRNA sprays currently in development for commercial use. Importantly, while *L. decemlineata* displays high sensitivity to dsRNA foliar sprays^12^, results from recent baseline studies in Europe demonstrate that susceptibility varies greatly with geographic location^16^. These observations, coupled with the notorious nature of *L. decemlineata* to develop insecticide resistance and the known plasticity of its genome^17^, suggest that the genetic potential for the evolution of resistance is already present.

We report selection of a geographically heterogeneous population of *L. decemlineata*, generated from field collections in nine US states, with dsRNA targeting the V-ATPase subunit A gene. Using adult emergence as the endpoint, selection resulted in > 11,100-fold resistance after approximately nine episodes of selection. Results from bioassays with larvae derived from crosses and backcrosses support that resistance is transmitted as an autosomal recessive trait and is polygenic. Resistant *L. decemlineata* were cross-resistant to a dsRNA for an alternative efficacious gene target, suggesting that target mutations per se are not involved in resistance. Evaluation of target transcript abundance upon dsRNA treatment in CEAS 300 demonstrated lack of gene silencing, thus confirming resistance. The dsRNA-resistant *L. decemlineata* was slightly more tolerant than the parental susceptible strain to the Cry3Aa insecticidal protein from *B. thuringiensis*, an active ingredient in biopesticides registered for use against *L. decemlineata*^18^ and expressed in transgenic potato plants with potential for commercialization^19^. Overall, these results demonstrate that *L. decemlineata* can develop high levels of resistance against insecticidal dsRNA when delivered using non-transgenic foliar applications. This information should help guide the design of resistance management tools, and the availability of a dsRNA-resistant *L. decemlineata* strain will allow future research to identify resistance genes.

## Results

### Selection for resistance to dsRNA

Bioassays with dsRNA targeting the V-ATPase subunit A transcript against larvae from the GC colony demonstrated high insecticidal activity with an estimated LC_95_ of 0.38 μg/ml, which was used as the initial selective concentration. Episodes of selection and corresponding observations are summarized in Materials and Methods and Table 1. A single adult survivor was recorded out of the 300 larvae from the GC colony used in the first selection episode, suggesting an estimated frequency of resistant individuals in the GC colony of 0.3%. The surviving adults from GC and from selection of a subpopulation of survivors from initial bioassay tests (BA-A) were pooled to form the “chronically exposed adult surviving” (CEAS) colony, which was then allowed to sib-mate without selection to produce an F2 generation. Larvae from this CEAS F2 generation were selected with the same concentration of dsRNA (0.38 μg/ml), resulting in higher adult survivorship (53.6%) compared to the first selection episode. Six subsequent selection episodes were performed with increasing dsRNA concentrations, from 1.11 μg/ml until reaching 300 μg/ml (Table 1). Clear evidence for resistance was obtained from the increased percentage of adult survivors when exposed to increasing dsRNA concentrations in each selection episode. Importantly, bioassays with the GC parental colony using 5.68, 9.41 and 300 μg/ml of dsRNA resulted in at least 86.7% mortality, including 100% mortality at both 9.41 and 300 ug/ml dsRNA (Table 1), confirming activity of the dsRNA and demonstrating that increased CEAS survival was due to resistance.

**Table 1:** Summary of steps and observations in selection of resistance to dsRNA targeting the V-ATPase subunit A gene in *Leptinotarsa decemlineata.*

Selection episode	Subpopulation selected	No	dsRNA (µg/ml)	Adult survival (%)
1	GC	300	0.38	0.3
	BA-A	300	0.38	3.7
2	CEAS	1095	0.38	53.6
3	CEAS 0.38	150	1.11	67.3
4	CEAS 1.11	165	1.88	58.2
5	CEAS 1.88	30	5.68	93.3
	GC	30	5.68	13.3
6	CEAS 5.68	30	9.41	73.3
	GC	30	9.41	0.0
7	CEAS 9.41	75	20	81.3
	GC	30	20	6.7
8	CEAS 20	105	30	84.8
	GC	30	30	3.4
9	CEAS 30	60	300	95.0
	GC	30	300	0.0

**Figure 1:**
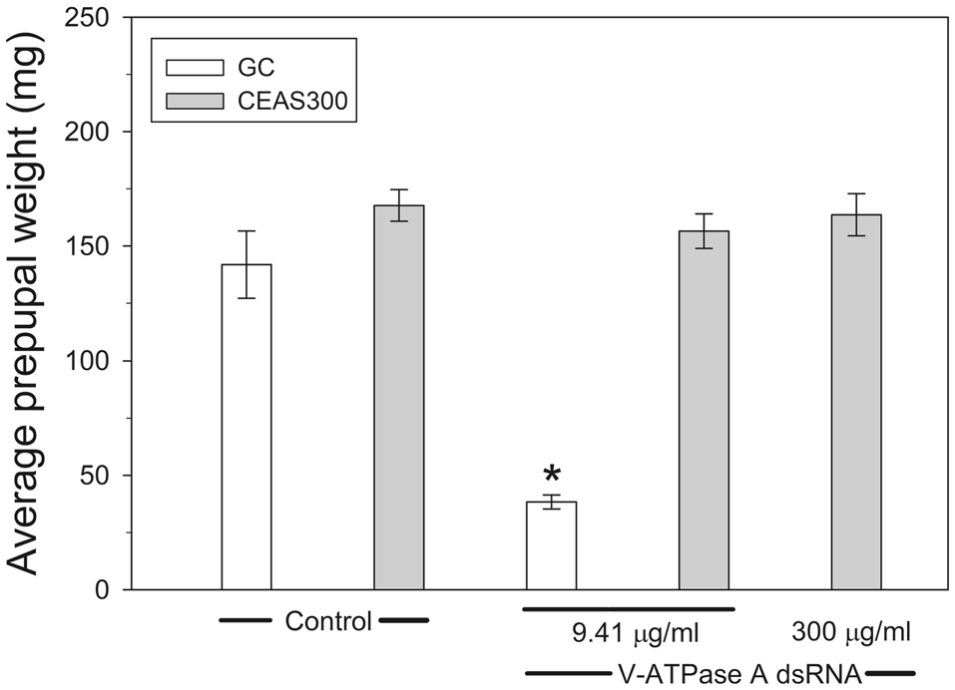
Average weight (in mg) of GC and CEAS prepupae when exposed to control, 9.41 μg/ml or 300 μg/ml of V-ATPase subunit A dsRNA throughout larval development. Asterisk denotes significant mean weight difference (*P* < 0.05, Mann–Whitney Rank Sum Test) between control and 9.41 μg/ml treatment in GC. No significant differences (*P* = 0.918, Kruskal–Wallis One Way ANOVA on Ranks) between any of the treatments in CEAS 300.

In each selection episode, a solution of dsRNA at the concentration stated was used to coat potato leaves, which were fed to *L. decemlineata* from second instar until prepupae. GC = general colony containing collections of *L. decemlineata* from locations detailed in Fig. 1, BA-A = colony of survivors from preliminary bioassays, CEAS = Chronic Exposure Adult Surviving (specific dsRNA dose to which the adults survived is stated in each selection step). No. = Number of larvae used in each episode of selection (for CEAS) or to determine control susceptibility to a dsRNA treatment (for GC).

Bioassays were conducted comparing adult emergence in CEAS 300 and GC when exposed to increasing concentrations of dsRNA. The LC_50_ estimated for the GC colony was 0.18 μg/ml (Table 2). In contrast, no significant mortality (*P* = 0.416, Mann–Whitney Rank Sum Test) was observed in CEAS 300 larvae treated with 2000 μg/ml, the highest dsRNA concentration tested (4.7% ± 2.2 mortality in treatment compared to 7.3% ± 2.3 in control). Based on these estimates, the CEAS 300 colony presents > 11,100-fold resistance to dsRNA when compared to the parental GC population. This extremely high level of resistance was also reflected when comparing prepupal weights between GC and CEAS 300 after treatment with dsRNA. As expected from its high susceptibility, GC prepupae presented significantly reduced weight (*P* < 0.05, Mann–Whitney Rank Sum Test) after treatment with 9.41 μg/ml of V-ATPase A dsRNA when compared with the control treatment (Fig. 1). In contrast, there were no significant differences (*P* = 0.918, Kruskal–Wallis One Way ANOVA on Ranks) in prepupal weights of CEAS 300 in control versus treated individuals at 9.41 μg/ml or even 300 μg/ml of V-ATPase A dsRNA (Fig. 1).

**Table 2:** Concentration–response bioassays of GC and CEAS 300 *Leptinotarsa decemlineata* strains to dsRNA targeting the V-ATPase subunit A gene (dsRNA) and Cry3Aa protoxin from *Bacillus thuringiensis* (Cry3Aa) in leaf bioassays.

Strain	Treatment	LC_50_^a^ (95% FL)	RR^b^	Slope ± SE	χ^2^ (df)^c^
GC	dsRNA	0.18 (0.09–0.29)	–	0.87 ± 0.11	19.589 (22)
	Cry3Aa	4.22 (1.49–8.70)	–	1.34 ± 0.23	10.612 (8)
CEAS 300	dsRNA	> 2000^d^	> 11,100	–	–
	Cry3Aa	17.57 (10.30–28.01)	4.2 (1.5–8.7)^e^	1.78 ± 0.38	5.086 (8)

^a^LC_50_ values expressed in μg/ml.

^b^Resistance Ratio, estimated for dsRNA as the ratio of the LC_50_ value for GC to the highest dose tested without mortality for CEAS 300 (2,000 µg/ml); and for Cry3Aa protoxin as the lethal concentration ratio estimated by probit analysis in the POLO-PLUS program.

^c^Chi square (χ^2^) value and degrees of freedom (df) from fitting the data to the probit model.

^d^No significant mortality when compared to controls was detected at 2,000 µg/ml (highest dsRNA dose tested).

^e^Resistance ratio and 95% confidence intervals (in parenthesis) calculated using POLO-PLUS as described in Materials and Methods.

### Detection of RNAi gene silencing

**Figure 2:**
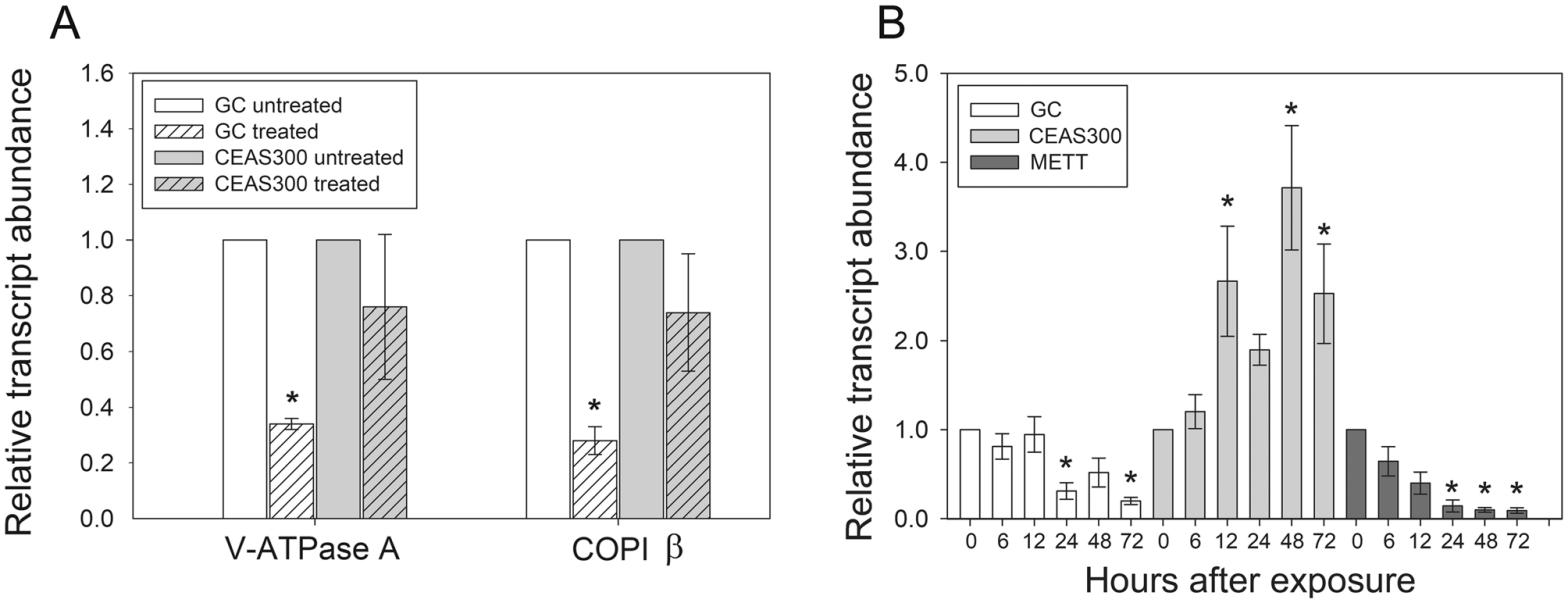
Change in target transcript abundance at different time points after feeding *Leptinotarsa decemlineata* larvae with potato leaves coated with dsRNA or a drop containing dsRNA. (**A**) Transcript abundance (relative to levels in larvae fed untreated potato leaves) in larvae from susceptible (GC) and dsRNA-resistant (CEAS 300) strains when fed potato leaves coated in a solution with 400 μg/ml of dsRNA targeting the V-ATPase subunit A transcript. Asterisks denote significant differences from untreated controls within each population (One Way Analysis of Variance followed by Holm-Sidak’s pairwise multiple comparisons test, *P* < 0.05). (**B**) Transcript abundance (relative to time 0 within each strain) when *L. decemlineata* larvae from susceptible (GC and METT) and dsRNA-resistant (CEAS 300) strains were treated with 25 μg of dsRNA targeting the V-ATPase subunit A transcript. Asterisks denote significant differences from time 0 (control) within each population (Kruskall-Wallis ANOVA on Ranks followed by a Dunnet’s pairwise multiple comparison to the control group, *P* < 0.05).

After a chronic exposure for 48 h to potato leaves coated with 400 μg/ml of dsRNA targeting the V-ATPase subunit A gene, larvae from the GC strain presented a significant (*P* < 0.05, Holm-Sidak’s pairwise multiple comparisons test) reduction in the levels of V-ATPase subunit A transcript (2.9-fold) when compared to larvae fed non-treated leaves (Fig. 2A). In contrast, no significant differences (*P* = 0.901, Holm-Sidak’s pairwise multiple comparisons test) in V-ATPase subunit A transcript levels were detected between control and treated larvae of the CEAS 300 strain (Fig. 2A).

Acute exposure of larvae from the GC population to a single dose of dsRNA targeting the V-ATPase subunit A gene (25 μg fed in a drop) resulted in a significant reduction in transcript levels 24 h (3.2-fold) and 72 h (5.0-fold) post-treatment (*P* < 0.05, Dunnett’s multiple comparison) (Fig. 2B). A two-fold reduction was also detected 48 h after treatment, but high variability in one of the six biological replicates led to a lack of significant differences from transcript levels at time 0 (*P* = 0.065, Dunnett’s multiple comparison). A similar pattern of transcript level reduction was observed in another susceptible population tested (METT), with transcript level reductions ranging from 11 to 16.4-fold that were all significantly different (*P* < 0.05, Dunnett’s multiple comparisons) from initial levels (Fig. 2B). Reductions in transcript levels at 24, 48 and 72 h compared to initial levels were not statistically different between GC and METT (*P* > 0.05, Tukey test for multiple comparisons). These results validated strain METT as an additional susceptible reference colony for comparisons with CEAS 300.

In contrast to results from susceptible strains, acute exposure (drop feeding) of larvae from the resistant CEAS 300 population resulted in a significant increase (*P* < 0.05, Dunnett’s multiple comparison) in abundance of the target V-ATPase subunit A transcript for at 12, 48, and 72 h. post treatment, reaching a maximum of 3.7-fold increase at 48 h post-treatment. (Fig. 2B).

### Cross-resistance to alternate dsRNA targets and Cry3Aa

**Figure 3:**
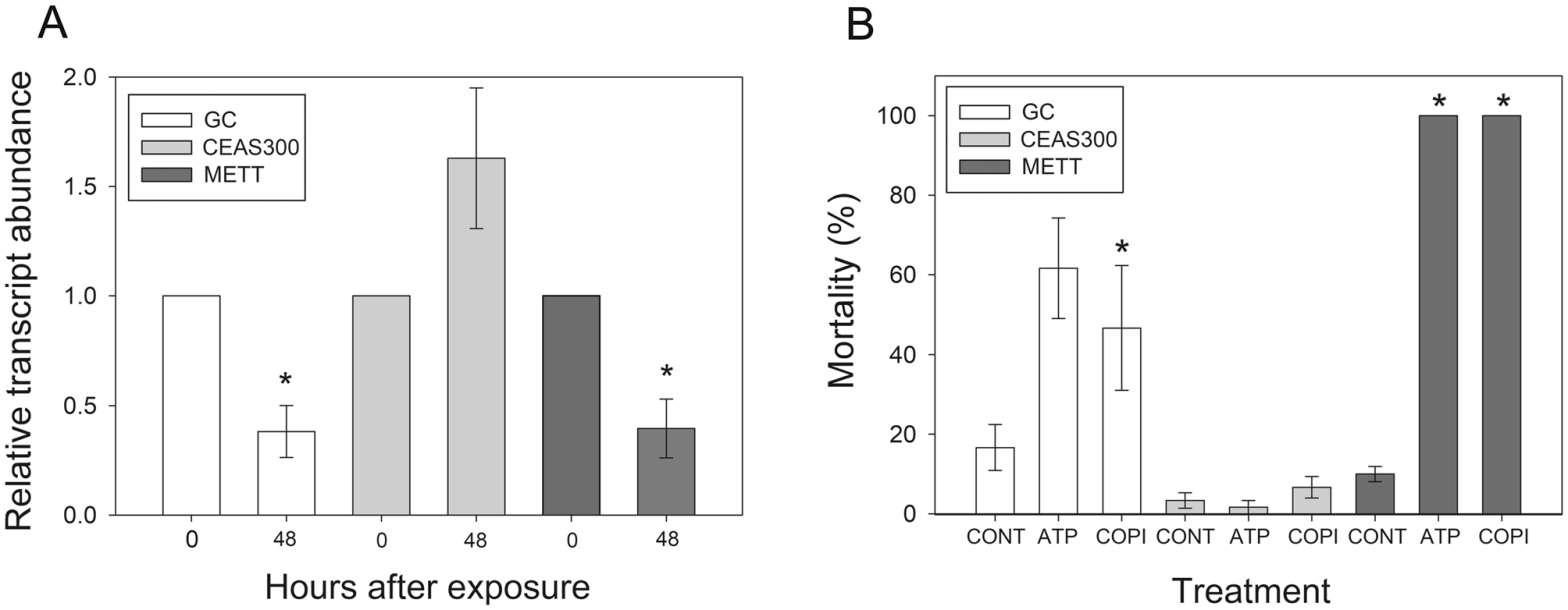
Cross-resistance to COPI β (coatomer subunit beta) dsRNA in CEAS 300. (**A**) Changes in target transcript abundance 48 h post-feeding on a drop containing 25 μg of COPI β dsRNA detected by qRT-PCR. Larvae from susceptible (GC and METT) and resistant (CEAS 300) *Leptinotarsa decemlineata* colonies were treated and their gut dissected after 48 h. Asterisks denote significant difference from time 0 within each population (t-test, *P* < 0.05). (**B**) Percentage mortality when larvae were exposed to 600 µg/ml of dsRNA targeting the V-ATPase subunit A (ATP) or COPI β (COPI) transcript compared to control (CONT) treatment during the larval stage. Mortality was assessed during adult emergence.

Before testing for cross-resistance to alternate dsRNAs in CEAS 300, we tested the change in target transcript abundance induced by treatment with an alternative dsRNA target, coatomer subunit beta (COPI β). Treatment with COPI β dsRNA delivered on potato leaves (Fig. 2A) or by drop feeding (Fig. 3A) resulted in significant reduction (*P* < 0.05, Holm-Sidak’s and Dunnett’s multiple comparison tests) in target transcript abundance in samples from the GC or METT populations after 48 h. This reduction in COPI β transcript levels in the GC and METT populations supported that COPI β dsRNA triggered the RNAi response and validated its use for testing cross-resistance in CEAS 300. In contrast, no significant differences in COPI β transcript levels were observed in larvae from the CEAS 300 strain after treatment with COPI β dsRNA (Holm-Sidak’s and Dunnett’s multiple comparison tests, *P* > 0.05) (Figs. 2A, 3A), suggestive of cross-resistance.

A single concentration bioassay (600 μg/ml) was performed using COPI β dsRNA for testing cross-resistance, using the same concentration of the V-ATPase subunit A dsRNA as a positive control. For both dsRNA targets, mortality in the susceptible populations, GC and METT, was significantly higher than in the CEAS 300 population (*P* < 0.05, Dunnett’s multiple comparison) (Fig. 3B). In GC, treatment with the dsRNA targeting the V-ATPase subunit A transcript or COPI β dsRNA caused 61.6% and 46.7% mortality, respectively. These mortality values were lower than expected for the GC colony based on previous bioassays (Tables 1 and 2). In comparison, mortality in the METT colony reached 100% for both dsRNAs, supporting that dsRNA activity was not affected. This observation suggested the possibility that the GC colony could have been contaminated with CEAS 300 individuals during routine rearing in the 10 month period between selection and testing for cross-resistance. In contrast, no significant differences were observed between mortality from control and treatment with V-ATPase subunit A dsRNA (*P* = 0.537, t-test) or COPI β dsRNA (*P* = 0.357, t-test) in CEAS 300, supporting cross-resistance to COPI β.

Cross-resistance to Cry3Aa protoxin was tested comparing susceptibility in larvae from the GC and CEAS 300 (with > 11,100-fold resistance to dsRNA) populations. Based on the estimated LC_50_ values (Table 2), a 4.2-fold resistance ratio (95% confidence intervals 2.0–8.8) was estimated between GC and CEAS 300. Hypothesis tests with probit regression lines rejected equality (*P* < 0.05) but not parallelism (*P* = 0.609), indicating that probit lines had equal slopes but different intercepts (Supplementary Fig. S1), suggestive of quantitative differences in enzymes affecting activity of the toxin^20^.

### Genetics of resistance to dsRNA

Individuals from the F1 generation from crossing GC × CEAS 300 were used in bioassays testing transmission of the resistance trait. There were no significant differences in susceptibility depending on the gender of the resistant parent in the cross (*P* = 0.208, t-test), supporting that resistance was not sex-linked or maternally inherited (Table 3). Consequently, in further analyses, data from crosses in which male or female CEAS 300 beetles were used as parents were pooled. Notably, we observed lower (56.6%) than expected (100%) susceptibility in replicated tests with 800 μg/ml of dsRNA against offspring from GC × GC crosses (Table 3), in line with the lower than expected susceptibility detected in cross-resistance bioassays. Therefore, we included tests with METT as an additional reference susceptible strain and confirmed that as observed for GC, resistance was not dependent on the gender of the resistant parent in the cross (*P* = 0.177, t-test), supporting autosomal resistance.

**Table 3:** Percentage mortality (± standard error, SE) observed in progenies from different crosses when exposed to 800 µg/ml of V-ATPase subunit A dsRNA in 20-day leaf-feeding bioassays. These mortalities were used to determine the genetics of resistance in CEAS 300.

Test	N^a^	Control mortality (%) ± SE	Corrected treatment mortality (%) ± SE
**F1 bioassay 1—GC and CEAS 300**			
GC (♀) × GC (♂)	4	1.7 ± 1.7	56.6 ± 10.0
GC (♀) × CEAS 300 (♂)	4	1.7 ± 1.7	31.7 ± 5.7
CEAS 300 (♀) × GC (♂)	4	1.7 ± 1.7	40.0 ± 7.7
CEAS 300 (♀) × CEAS 300 (♂)	4	1.7 ± 1.7	1.7 ± 1.7
**F1 bioassay 2—METT and CEAS 300**			
METT (♀) × METT (♂)	8	11.7 ± 4.8	100.0 ± 0.0
METT (♀) × CEAS 300 (♂)	4	23.4 ± 1.9	95.6 ± 3.3
CEAS 300 (♀) × METT (♂)	8	8.3 ± 2.1	94.0 ± 2.1
CEAS 300 (♀) × CEAS 300 (♂)	8	4.2 ± 1.7	7.7 ± 1.7
**Backcross bioassay—METT, CEAS 300 and F1**			
METT (♀) × METT (♂)	8	6.6 ± 4.2	98.0 ± 1.7
METT (♀) × F1^b^ (♂)	8	10.8 ± 4.5	73.7 ± 7.2
F1 (♀) × METT (♂)	4	6.7 ± 2.7	96.4 ± 1.9
CEAS 300 (♀) × F1 (♂)	4	0.0 ± 0.0	36.6 ± 4.3
CEAS 300 (♀) × CEAS 300 (♂)	4	5.0 ± 3.2	13.3 ± 9.4

^a^Number of biological replicates performed, each consisting of 15 randomly selected larvae.

^b^F1 generation resulting from crossing METT and CEAS 300.

The value of dominance (*h*) was calculated to determine if resistance was transmitted as a recessive (*h* = 0), incompletely recessive (*h* between 0 and 0.5), or dominant (*h* = 1) trait. The estimated degree of dominance for offspring from the GC × CEAS 300 crosses was *h* = 0.37, while for the METT × CEAS 300 it was *h* = 0.06. The *h* value from GC × CEAS 300 was probably influenced by the lower susceptibility to the dsRNA detected in the GC strain . Therefore, results with METT × CEAS 300 agreed with those from GC × CEAS 300 crosses and supported the conclusion that resistance is transmitted as an incompletely recessive trait. Based on these observations, only the METT strain was used in subsequent backcrossing experiments to determine the number of loci involved.

Backcrosses of the F1 generation from METT × CEAS 300 crosses (F1_METTxCEAS300_) to the susceptible METT (BC1 backcross) or resistant CEAS 30 (BC2 backcross) strains were used in testing for monogenic versus polygenic resistance. Survival of larvae after exposure to dsRNA was used in the Chi-square (χ^2^) goodness-of-fit model with monogenic resistance as the null hypothesis. Significant differences (*P* < 0.05) were detected between the observed and expected mortalities of both backcrosses. The estimated Σχ^2^ value for BC1 was 415.6, while for BC2 it was 9.09, both of which are significantly higher than the tabulated $\chi$^2^ value of 3.84 at the 0.05 significance level and 1 degree of freedom. These results support that resistance to dsRNA in CEAS 300 involves multiple loci.

## Discussion

The sustainable use and long-term efficacy of dsRNA-based insecticidal products should rely heavily on effective insect resistance management (IRM) strategies guided by information obtained from resistance studies in model pests. Using *L. decemlineata*, a target pest for development of dsRNA-based insecticidal products and a species with a history of developing resistance to nearly every class of insecticide^15^, we describe the first case of insect resistance against non-transgenic foliar delivery of insecticidal dsRNA. The relatively quick and very high levels of resistance observed (> 11,100-fold) were probably, in part, a result of our selection protocol. For instance, we used *L. decemlineata* collections from diverse locations in the USA to generate a geographically heterogeneous parental *L. decemlineata* population. Recent reports showing genetic differences between *L. decemlineata* populations from the northwestern and eastern USA influencing resistance to insecticides^21^, support that our strategy probably also generated a genetically-diverse parental population. In addition, we used chronic exposure through the larval stage and adult emergence as the endpoint to select for major resistance alleles allowing completion of the entire life cycle after exposure to the dsRNA. The foliar coating method of dsRNA delivery also allowed the use of increasing concentrations of dsRNA that are more difficult to achieve with plants expressing dsRNA, as was the case for *D. v. virgifera*. These characteristics of the selection process probably contributed to relatively quick selection of larvae carrying major resistance alleles.

A recent baseline study demonstrated variation in dsRNA sensitivity (7.5-fold difference between the most and least susceptible populations) among *L. decemlineata* populations collected from 14 locations across Europe^16^. In addition, the genome of *L. decemlineata* contains a high proportion of single nucleotide polymorphisms (SNPs) and transposable elements, contributing to high plasticity^17^. Taken together with results from this study, these observations not only demonstrate the genetic potential for resistance development against dsRNA delivered through a non-transgenic foliar application in *L. decemlineata*, they also suggest individuals carrying resistance alleles may not be rare in field populations. Both targeted screening for resistance genes once identified, and F1 screens, would help to establish the frequency of resistance alleles to dsRNA in field populations of *L. decemlineata*.

Results from RT-qPCR demonstrated a lack of gene silencing in CEAS 300 gut tissues and detected no change (chronic exposure) or a significant increase (acute exposure) in the transcript abundance of the target gene. A previous study demonstrated that lack of gene silencing in dsRNA-resistant *D. v.* virgifera could be equally detected using gut tissues or carcasses^8^. Additional experiments conducted on different tissues could be of value in further identifying the mechanism of resistance to dsRNA in CEAS 300. Interestingly, increased target transcript levels were only detected in CEAS 300 gut tissue after acute exposure to a high dose of V-ATPase A dsRNA (25 μg) but not during chronic exposure by feeding for 48 h on potato leaves coated with a lower dsRNA concentration (0.4 μg/μl). Although there was a trend for higher target transcript levels, no significant differences were detected in CEAS 300 larvae challenged with a drop containing 25 μg of COPI β dsRNA. Though up-regulation of the target gene has been previously observed after treatment with dsRNA^22–24^, the mechanism for this is unknown. A previous study reported up-regulation of V-ATPase subunit E in *L. decemlineata* after treatment with pesticides^25^ similar to the up-regulation of V-ATPase subunit A observed after dsRNA treatment in CEAS 300. It is plausible that up-regulation of V-ATPase subunits is involved in stress response in *L. decemlineata* exposed to pesticides and dsRNA. However, because this target up-regulation was not observed under the leaf-feeding conditions used for bioassays, it may be a phenomenon associated with acute exposure to high concentrations of dsRNA in CEAS 300 rather than the main mechanism of resistance to dsRNA in that strain. Further experimentation would be needed to determine if and how this up-regulation is associated with resistance.

The > 11,100-fold dsRNA-resistant *L. decemlineata* colony showed only ~ four-fold reduced susceptibility against Cry3Aa protoxin, with no significant difference in mortality between the susceptible and resistant *L. decemlineata* populations found (Mann–Whitney Rank Sum Test, *P* = 0.333) at the Cry3Aa protoxin concentration causing 90% mortality in GC (50 µg/ml). It is plausible that combining *L. decemlineata* collections from thirteen different locations across the USA brought varying levels of susceptibility to Cry3Aa among individuals in the GC colony^21^. In support of this hypothesis, >6-fold differences in susceptibility to Cry3Bb1 were detected when comparing field populations of *D. v. virgifera* from diverse locations in the USA^26^. The highly stringent selection protocol used to generate the CEAS population likely selected for robust individuals that are slightly more tolerant to Cry3Aa^7^. Lack of cross-resistance to Cry3Aa in CEAS 300 mirrors results reported for DvSnf7 dsRNA-resistant *D. v. virgifera*^10^. These results are also in line with independent action of dsRNA and Cry3Bb1 shown in *L. decemlineata* and *D. u. howardi*^27^ and lack of cross-resistance to dsRNA in Cry3Bb1-resistant *D. v. virgifera*^7^. Overall, these observations suggest that pyramiding of dsRNA and Cry proteins as insecticidal compounds with different modes of action will help delay the evolution of resistance.

While resistance to dsRNA in *D. v. virgifera* was located on a single-locus^8^, resistance in CEAS 300 was polygenic. This observation is expected from a colony that has been selected with increasing concentrations of dsRNA, as multiple loci contributing to resistance may have been consecutively added during selection to an initially selected major resistance allele. In *D. v. virgifera* resistance was linked to reduced uptake of dsRNA in the midgut cells^8^. Current work in our group is focused on using genomic approaches to identify gene(s) involved in resistance to dsRNA in CEAS 300 and determining if impaired uptake of dsRNA is a common mechanism of resistance to dsRNA in chrysomelids.

Here, we provide a second example of a dsRNA-resistant insect colony and the first example of selection using a non-transgenic (i.e. foliar coating) dsRNA delivery method. The information from this study serves as a basis to build effective IRM strategies for non-transgenic RNAi (dsRNA sprays) and complements findings from resistance studies using transformative RNAi (transgenics). Though some of the general characteristics of resistance in *L. decemlineata* and *D. v. virgifera* are similar (mode of inheritance, cross-resistance to alternative dsRNA targets, lack of cross-resistance to Cry proteins), there are also differences with the level and potential mechanism(s) of resistance. This suggests that resistance to dsRNA might develop differently depending on the insect species and delivery method. Consequently, studies conducted on different insects and using different selection regimes should provide a more comprehensive picture. In this regard, it would also be informative to study resistance to dsRNA in an insect species not considered to develop resistance to pesticides easily. This will help ascertain if a common resistance management framework is possible for different types of dsRNA delivery technologies and different target insects.

## Methods

### Insect colonies

The study complied with relevant institutional, national, and international guidelines and legislation. Adults, eggs, and larvae of *L. decemlineata* were collected from nine U.S. states, including Maine, Minnesota, New York, North Carolina, Oregon, Pennsylvania, Tennessee, Washington, and Wisconsin, for a total of thirteen locations (Supplementary Fig. S2). Approximately 300 adults and 25 larvae were collected at each location. Collection packages were returned to the University of Tennessee, where eggs and larvae were reared to adults on potato plants and then pooled with adult insects from the same location. This process continued for six months with multiple collections per location, and then adults from each subpopulation were pooled to generate the GC (General Colony) population. The GC population was reared for approximately 1 year (about 12 generations), using methods described below, to reach a size of 1700 individuals before being used for bioassays and selection. Another dsRNA-susceptible colony, METT, was generated from adults received from the University of Maine. Initial tests suggested this strain presented slight susceptibility to 0.1% Tween-20 (a wetting agent used in bioassays) and had to be adapted by rearing on potato leaves coated with 0.2% Tween-20 over three generations before use in bioassays.

Beetles were maintained in aluminum screened cages (61 cm tall and wide, 122 cm depth) in a greenhouse bay maintained at 27–30 °C, > 70% RH, 18L:6D photoperiod, and fed potato plants (*Solanum tuberosum* var. Desiree) ad libitum in 11.3 L (3 gallon) containers. Potato plants were grown pesticide-free using natural enemies to control other arthropod pests whenever required, in dedicated greenhouses at the University of Tennessee AgResearch Plateau Research and Education Center (Crossville, TN), and were only used when disease-free (based on visual inspection). Eggs were collected from potato plants every two days and kept until hatching (4–5 days) in clear plastic cups with perforated lids to allow air circulation. All life stages from egg to adult were maintained in an incubator (Percival, Perry, IA) set at 26 °C, 75% RH, and 18L:6D photoperiod. Larvae hatching on the same day were fed ad libitum on potato leaves in 150 mm Petri dishes with a two-inch diameter hole cut in the lid covered with a screen mesh (1 × 1 mm). After 96 h, larvae were moved to 1-L plastic containers covered with a lid with 2-cm diameter holes covered with an aluminum mesh, to continue feeding ad libitum. After day 6 of exposure, larvae were transferred to new containers every 24 h to reduce accumulation of nitrogenous toxic waste. When reaching the prepupal stage (8–10 days), prepupae were placed in pupation chambers—2L plastic containers half-filled with potting soil (Pro-mix Bx Bio-fungicide + Mycorrhizae)—lightly sprayed with distilled water. Untreated potato leaves were laid on the surface of the soil until all larvae reached the prepupal stage and burrowed into the soil. Pupae remained in pupation chambers until adult emergence (5–6 days) when they were put in rearing cages for mating and oviposition.

### Bioassays

Chronic larval toxicity bioassays were conducted using lethality at adult emergence as the endpoint, which was scored approximately 20 days after initiation of the bioassay. Second instar *L. decemlineata* were exposed to potato leaves covered in a solution of a previously described^1^ dsRNA targeting the *L. decemlineata* V-ATPase subunit A (generously provided by Monsanto, St. Louis, MO, Supplementary Table 1) in 0.1% Tween-20 as a wetting agent. Seven dsRNA concentrations were initially tested, including 0.01, 0.03, 0.09, 0.18, 0.27, 0.54, and 0.81 μg/ml, and water with 0.1% Tween-20 was used as a control. Freshly cut potato leaves were prepared by submerging in the dosing solution for approximately 10 s, after which the petiole was inserted into a tube containing distilled water to maintain turgidity while it was allowed to air dry completely (15–20 min) on metallic racks^13^. Fifteen randomly-selected second instars per 150 mm Petri dish were evaluated (considered a biological replicate), with a minimum of four replicates performed per dsRNA concentration. Larvae fed ad libitum on the treated leaves, with new treated leaves added every 24 h. Rearing proceeded as described above until adult emergence. Time required to reach the prepupal stage and prepupa weights was recorded. Prepupal weights were tested for significant differences using the Mann–Whitney Rank Sum Test (when comparing 2 samples) and the Kruskal–Wallis One Way ANOVA on Ranks test (when comparing 3 samples), both at the *P* < 0.05 significance level. The number of emerging adults was recorded daily, and mortality data were analyzed using POLO-PLUS (LeOra Software Company) to estimate lethality parameters^28^. Only bioassays with control mortality < 25% were considered.

### Selection for resistance to dsRNA

The bioassay protocol described above was used for selection of larvae from the GC colony for resistance to dsRNA targeting the V-ATPase subunit A transcript. The selection process and the observed adult survivorship results are shown in Table 1. The first episode of selection included 300 individuals from the GC colony and larvae from a sub-colony (BA-A) created from insects from the GC colony that survived preliminary bioassays estimating the dsRNA selection concentration. The first episode of selection used 0.38 μg/ml as the lethal concentration of dsRNA killing 95% of the individuals (LC_95_) from preliminary bioassays with GC, using adult emergence as an endpoint. Selection proceeded as described for bioassays above, with 15 larvae per container, for a total of 300 larvae each from the GC and the BA-A survivor subcolony. Adults surviving selection, 1 from GC and 11 from BA-A, were pooled to generate the chronically exposed adult survivors (CEAS) colony. Adults in the CEAS colony were sib-mated in aluminum screened cages in a greenhouse bay maintained at 27–30 °C, > 70% RH, 18L:6D photoperiod, for two consecutive generations to increase colony size and preserve potential recessive resistance alleles, and then larvae from the F2 generation were used in the second selection episode (Table 1). Fifteen larvae from the same egg clutch were used per container, with 73 containers (1095 larvae) fed potato leaves coated with 0.38 μg/ml of dsRNA. Survivors (587, 53.6%) were sib-mated to generate the CEAS 0.38 subpopulation. Larvae from CEAS 0.38 were used in the third episode of selection using 1.11 μg/ml of dsRNA. Survivors (101, 67.3%) were sib-mated to generate the CEAS 1.11 subpopulation. This process of generating a new subpopulation from adult survivors after exposure to increasing concentrations of dsRNA was performed using selection with 1.88, 5.68, 9.41, 20, 30 and 300 μg/ml of dsRNA (Table 1). Adult survivors from exposure to 300 μg/ml of dsRNA (57, 95%) were pooled to generate the CEAS 300 colony, which was used for concentration bioassays (same protocol as bioassays described above) estimating the levels of resistance, and for resistance characterization. Bioassays with the parental GC colony were performed simultaneously and with the same dsRNA concentration as episodes of selection 5, 6 and 9 in Table 1, as controls to determine that increased survival in CEAS was not due to lack of activity of the dsRNA used for selection. The CEAS 300 colony was maintained under constant selection during the larval stage with 400 μg/ml of dsRNA targeting the V-ATPase subunit A gene to preserve resistance alleles during performance of all experiments described in this manuscript.

### Testing for RNAi response

Larvae from susceptible (GC or METT) and resistant (CEAS 300) *L. decemlineata* populations were reared on untreated potato leaves until treatment with dsRNA. Tests were performed using early fourth instar larvae reared on untreated potato leaves and starved for 24 h before exposure to dsRNA. In tests with plant tissue, potato leaves were coated with a solution of 400 μg/ml of dsRNA targeting the V-ATPase subunit A gene or COPI β (coatomer subunit beta; generously provided by Monsanto, St. Louis, MO, Supplementary Table 1). Larvae were allowed to feed on dsRNA-treated or non-treated potato leaves as a control for 48 h, and then guts were dissected and used for RNA purification. Drop feeding tests were performed by feeding a 5 μl droplet of a 1 mg/ml sucrose solution (to promote ingestion) containing 25 μg of dsRNA targeting the V-ATPase subunit A gene or COPI β dsRNA. Larvae were closely monitored to ensure they completely consumed the droplet, and then they were transferred to untreated potato leaves until gut dissection at 0, 6, 12, 24, 48, or 72 h post-drop-feeding.

Total RNA was isolated from a pool of 3 dissected guts (one biological replicate) using the RNeasy Mini kit (Qiagen), according to manufacturer’s specifications. Purified total RNA (2 µg) was used to synthesize cDNA with random hexamer primers using the PrimeScript 1st strand cDNA Synthesis Kit (Takara), according to manufacturer’s instructions. Quantitative PCR assays included 3 μl of 10 × diluted cDNA, 10 μl of master mix (PerfeCTa qPCR FastMix II, Low ROX, QuantaBio), 0.5 μM of each primer, 0.25 μM probe and 4.5 μl of nuclease-free water, for a total volume of 20 μl. The thermocycler conditions included one denaturation cycle at 95 °C for 2 min, followed by 40 cycles of denaturation at 95 °C for 10 s and annealing/extension at 60 °C for 30 s. Transcript levels for target genes were estimated by the 2^−ΔΔCT^ method^29^ using the *RP18* (GenBank accession: KC190034) housekeeping gene for normalization^30^. Mean transcript levels and corresponding standard errors were calculated from three independent biological replicates tested in triplicate for each target gene. Transcript levels were expressed relative to time zero (dissected immediately after dsRNA consumption) or untreated samples being considered as a relative level of “1”. One Way Analysis of Variance (ANOVA) followed by Holm-Sidak’s pairwise multiple comparisons (leaf-feeding tests) or a Kruskall-Wallis ANOVA on Ranks followed by a Dunnet’s pairwise multiple comparison to a control group (drop feeding) at the *P* < 0.05 significance level were used to determine significant differences, comparing transcript levels in the treated and control samples.

### Cry3Aa protoxin

An isolated colony of *B. thuringiensis* subsp. *morrisoni* biovar *tenebrionis* (*Bacillus* Genetic Stock Center, Columbus, OH) from a 1/3 tryptic soy broth (TSB) plate was suspended in autoclaved water and used to inoculate flasks with 1/3 TSB medium. The cultures were incubated for 3 days at 28 °C and 160 rpm agitation until sporulation was confirmed by microscopic observation. Cultures were then centrifuged at 10,000 rpm for 10 min and pellets washed with 1 M NaCl containing 0.1% Triton X-100 and recovered by centrifugation, and this process was performed thrice followed by three washes with distilled water. The final pellet was resuspended in solubilizing solution (50 mM Na_2_CO_3_, 0.1% β-mercaptoethanol, 0.1 M NaCl) and incubated overnight at 30ºC and 200 rpm. The solubilized solution was centrifuged at 14,500 rpm for 30 min to pellet spores, and the supernatant was loaded on a HiTrap Q HP column pre-equilibrated in 50 mM Na_2_CO_3_, pH 9 (buffer A) for purification by anion exchange in an AKTA FPLC system (GE Healthcare). Elution was performed using a linear gradient of buffer B (buffer A containing 1 M NaCl). Fractions containing a band of the expected Cry3Aa protoxin size (~ 73 kDa) were identified by SDS-10%PAGE, and then pooled, quantified^31^ with BSA as standard, and kept at -80 °C until use.

### Evaluating for potential cross-resistance to an alternate dsRNA and Cry3Aa

Bioassays to determine cross-resistance between CEAS and either an alternate dsRNA or Cry3Aa were performed as described above. Eggs were collected from different batches for all replicates to ensure genetic variability, and four (dsRNA) or two (Cry3Aa) biological replicates with 15 larvae each were performed for each concentration. Mortality was estimated based on unsuccessful adult emergence for each treatment. Only bioassays with control mortality < 25% were considered.

An additional dsRNA target (COPI β; coatomer subunit beta, Supplementary Table 1) distinct from the V-ATPase subunit A gene targeted during selection, was used to test for cross-resistance. RNAi-induced silencing of COPI β was previously shown to cause high mortality in *L. decemlineata*^32^*.* Neonates were fed on non-treated potato leaves until reaching second instar, and then larvae were chronically exposed to potato leaves dipped in a single discriminatory concentration (600 μg/ml) of dsRNA targeting the V-ATPase subunit A transcript or COPI β dsRNA in 0.1% Tween-20 as a wetting agent. This discriminatory dsRNA concentration was chosen as the concentration of dsRNA targeting the V-ATPase subunit A transcript resulting in 100% mortality of GC and METT individuals while not affecting performance of insects from the CEAS 300 strain. Leaves dipped in 0.1% Tween-20 were used as a control.

Cross-resistance to Cry3Aa protoxin was tested by exposing susceptible (GC) and CEAS 300 *L. decemlineata* second instar larvae to potato leaves dipped in solutions containing increasing Cry3Aa protoxin concentrations (0.5, 2, 5, 20 and 50 µg/ml) in 0.1% Tween-20 as wetting agent. Larvae fed on leaves dipped in 0.1% Tween-20 were used as a control. After 48 h of exposure, larvae were transferred to containers with toxin-free potato foliage to complete development. The POLO-PLUS software^28^ was used to conduct probit analysis (Fig. S1) and determine the LC_50_ value for the tested populations. Lethal concentration ratios and corresponding 95% confidence limits were estimated from LC_50_ values in POLO-PLUS, as described elsewhere^20^. Tests for equality and parallelism in probit regression lines were examined (rejected if *P* < 0.05) using a log-likelihood ratio test from chi-square statistics, calculated by POLO-PLUS, and the degrees of freedom.

### Evaluating inheritance of resistance to dsRNA

Crosses were set up between adults from susceptible (GC and METT) and dsRNA-resistant (CEAS 300) strains of *L. decemlineata* to determine the inheritance of resistance to dsRNA. Pupae from each strain were sexed^33^ and kept separated by gender in 2-L plastic containers. When adults emerged, they were fed potato leaves until they became sexually mature (approximately two weeks). Crosses used ten adults of each sex from each parental strain, and they were placed in a bug dorm (BioQuip Products Inc., Compton, CA) with a single potato plant in a 11-L pot for feeding and oviposition. Plants were changed as needed after 3–5 days. Eggs were harvested three days after cross initiation and neonates maintained on untreated potato leaf tissue until reaching 2nd instar. Larvae were then used for bioassays with a single discriminatory dsRNA concentration (800 µg/ml) known to kill 100% of susceptible larvae but no larvae from the CEAS 300 strain. The bioassay protocols were as described above.

Abbott’s formula was used to calculate corrected mortality when control mortality was greater than 5%^34^, and only bioassays with control mortality < 25% were considered. Results from these bioassays (Table 3) were used to estimate the degree of dominance (*h*), using the single-concentration method, as described elsewhere^35^. According to this method, dominance (*h*) is estimated as *h* = (w_12 _− w_22_)/(w_11 _− w_22_), where w_11,_ w_12_ and w_22_ are the fitness of resistant homozygotes, heterozygotes, and susceptible homozygotes, respectively, at the tested dsRNA concentration. The value of *h* ranges from 0 for completely recessive to 1 for completely dominant. The fitness of treated resistant homozygotes was considered as 1, while fitness of susceptible homozygotes and heterozygotes was estimated by dividing their survival rate by the survival rate of resistant homozygotes. The survival rate was estimated as (100%—mortality %) at each dsRNA concentration.

To determine the number of genes involved with dsRNA resistance, two sets of backcrosses were conducted by mating adults from the F1 generation resulting from CEAS 300 × METT crosses with male or female beetles from the susceptible (METT, BC1 backcross), or female beetles from resistant (CEAS 300, BC2 backcross) parental strains. Larvae from both backcrosses were exposed to 800 μg/ml of dsRNA targeting the V-ATPase subunit A transcript, using the bioassay protocols described above. Results of these bioassays (Table 3) were used to estimate the number of genes associated with resistance. A chi-square (*χ*^2^) goodness of fit was performed to test the null hypothesis of monogenic resistance, following the methods of Sokal and Rohlf^36^. The theoretical value of Σχ^2^ was calculated χ^2 ^= (*N*_*i*_ − *pn*_*i*_)^2^/*pqn*_*i*_, where *N*_*i*_ is the observed number of deaths in the backcross generation at the given dsRNA concentration, *p* is the expected mortality in the backcross generation estimated as described by Georghiou^37^, and *q* = 1 − *p.* The null hypothesis (monogenic resistance) was rejected if the calculated value of Σχ^2^ was greater than the table value of Σχ^2^ at the 0.05 significance level and 1 degree of freedom.

## Supplementary Information

Supplementary Information
