# Supplementary material for: Selection for high levels of resistance to double-stranded RNA (dsRNA) in Colorado potato beetle (Leptinotarsa decemlineata Say) using non-transgenic foliar delivery
Source: Sci Rep. 2021 Mar 22;11:6523. doi: 10.1038/s41598-021-85876-1 (PMC7985369; doi:10.1038/s41598-021-85876-1)
Supplement: Supplementary file 1 — Supplementary Information [file 41598_2021_85876_MOESM1_ESM.docx]

**Selection for high levels of resistance to double-stranded RNA (dsRNA) in Colorado potato beetle (*Leptinotarsa decemlineata* Say) using non-transgenic foliar delivery**

Swati Mishra, James Dee, William Moar, Jodi Dufner-Beattie, James Baum, Naymã Pinto Dias, Andrei Alyokhin, Aaron Buzza, Silvia I. Rondon, Mark Clough, Sandy Menasha, Russell Groves, Justin Clements, Ken Ostlie, Gary Felton, Tim Waters, William E. Snyder and Juan Luis Jurat-Fuentes

**Supplementary Information**

**Figure S1**: Probit dose-response curves for dsRNA targeting the V-ATPase subunit A gene (A) and Cry3Aa protoxin (B) against GC (red line) and CEAS 300 (blue line) populations, as determined by the POLO-PLUS software. No significant mortality was detected in CEAS 300 at the highest concentration tested (2,000 μg/ml of dsRNA), explaining the lack of a probit dose curve in (A).


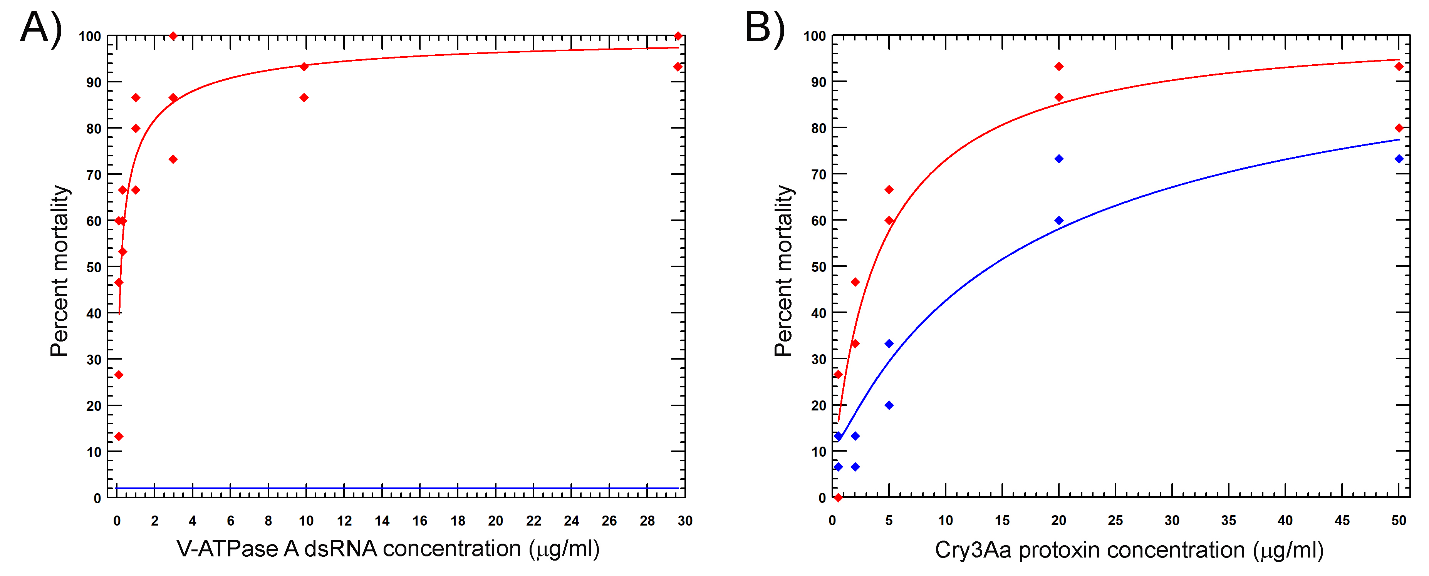


**Figure S2**: Collection states (grey) and sites for *Leptinotarsa decemlineata* used to generate the GC superpopulation.


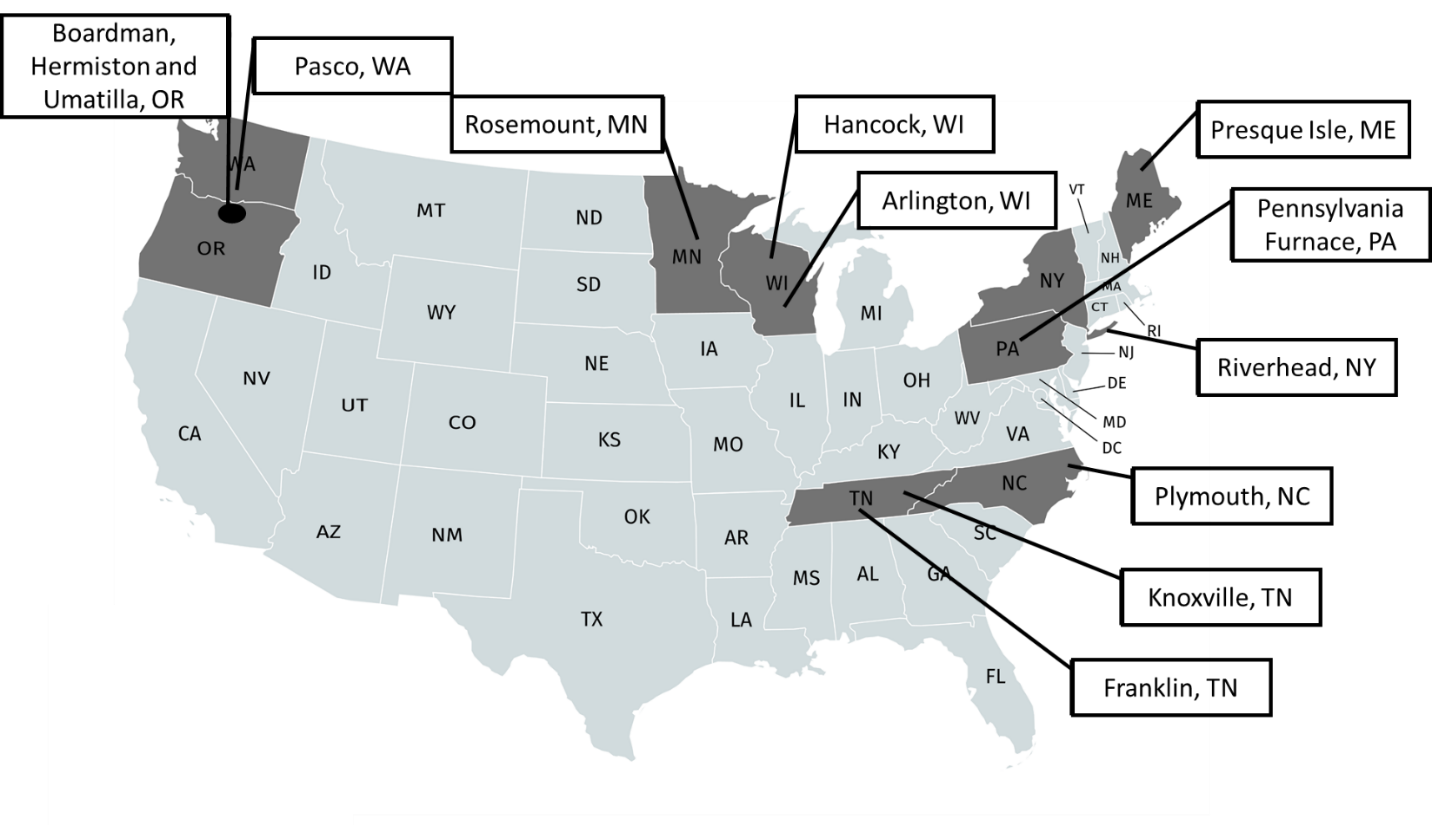


**Table S1**: Targeted *Leptinotarsa decemlineata* transcript, size and sequence of the dsRNAs used in the study.

| **Target** | **Size (bp)** | **Sequence** |
| --- | --- | --- |
| V-ATPase subunit A | 302 | GGUGACAUGGCCACCAUCCAGGUAUAUGAAGAAACUUCUGGAGUAACGGUGGGAGAUCCUGUGUUGCGUACCGGUAAACCUCUAUCUGUGGAACUUGGGCCAGGUAUUAUGGGUUCCAUCUUUGAUGGUAUCCAACGUCCGCUGAAAGACAUCUGCGACAUGACGGAAAGUAUCUACAUUCCCAAGGGUGUGAACGUGCCUUCACUCUCCAGAACUAUCAAAUGGGAAUUCAACCCAAUCAACAUCAAGUUGGGAUCCCACUUGACAGGUGGAGAUAUUUAUGGUAUGGUCCACGAAAACAC |
| Coatomer subunit beta (COPI β) | 300 | GUAAUCUGAAAAUGACUACCAUGGAACAGCCUUGUUACACACUCAUCAAUUUUCCGACAGAUUCUGAACCAUAUAAUGAAAUGCAACUCAAAACGGAUUUAGAGAAGGGCGAUGUGAAAGUCAAAAUACGAGCCUUAGAAAGAACCAUUCACAUGAUUUUGGCAGGAGAACGCCUGCCGAACGGUUUCUUAAUGACGAUUAUCAGGUAUGUUCUGCCGAUUCAGGAUCAUUUGGCCAAAAAGCUGUUGUUAAUUUUUUGGGAAAUUGUGCCAAAGACCUCACCUGACGGAAAACUGCUAC |
